# Drugs Prescribed for Asthma and Their Adverse Effects on Dental Health

**DOI:** 10.3390/dj11050113

**Published:** 2023-04-26

**Authors:** Edisson-Mauricio Pacheco-Quito, Jessica Jaramillo, Jéssica Sarmiento-Ordoñez, Katherine Cuenca-León

**Affiliations:** 1 Academic Unit of Health and Wellness, Faculty of Dentistry, Catholic University of Cuenca, Cuenca 010105, Ecuador; 2 Innovation and Pharmaceutical Development in Dentistry Research Group, Faculty of Dentistry, Head of Research and Innovation, Catholic University of Cuenca, Cuenca 010105, Ecuador; 3 Ministry of Health, Dentistry Department, Cuenca 010105, Ecuador

**Keywords:** asthma, drug therapy, dental health

## Abstract

Asthma is a chronic, heterogeneous respiratory pathology characterized by reversible airway inflammation. Therapeutics focus on symptom reduction and control, aimed at preserving normal pulmonary function and inducing bronchodilatation. The objective of this review is to describe the adverse effects produced by anti-asthmatic drugs on dental health, according to the reported scientific evidence. A bibliographic review was carried out on databases, such as Web of science, Scopus, and ScienceDirect. Most anti-asthmatic medications are administered using inhalers or nebulizers, making it impossible to avoid contact of the drug with hard dental tissues and oral mucosa, and thus promoting a greater risk of oral alterations, mainly due to decreases in the salivary flow and pH. Such changes can cause diseases, such as dental caries, dental erosion, tooth loss, periodontal disease, bone resorption, as well as fungal infections, such as oral candidiasis.

## 1. Introduction

Asthma is a heterogeneous disease, usually characterized by a chronic inflammatory disorder of the airways, elevated hyper-reactivity of the tracheobronchial tree, increased mucus production, and by symptoms, such as wheezing, coughing, chest tightness, and dyspnea, that can vary over time and in intensity, affecting people of both sexes, of all ages, and all races [1,2,3,4].

In recent years, its prevalence has increased significantly in most countries, with high rates of morbidity in children and mortality in adults, affecting more than 300 million people worldwide [5,6,7]. Prior to adolescence, the prevalence of asthma is higher in men than in women, but this tends to reverse after adolescence, because hormone levels in many women of childbearing age and the use of contraceptives can directly interfere with the treatment of asthma [8,9]. Mortality has increased significantly in recent years. According to the World Health Organization (WHO), the world death toll was estimated at 416,000 in 2016, mainly affecting developing countries [10]. More than 80% of these deaths have occurred in low- and middle-income countries [11,12,13]. 

The cause of asthma is not known, but there are risk factors that have been identified and gene-environment interactions. Genetics play a fundamental role in asthma; large genetic studies have identified hundreds of genetic variants associated with an increased risk of asthma [14,15]. The heritability of asthma ranges from 35% to 95% [16]. Other risk factors include allergic stimuli, such as house dust mites, animals, mold, and pollen. Non-allergic stimuli include viral infections, tobacco smoke, and cold air [3,17,18]. 

Diagnosis is made by clinical evaluation of symptoms, such as dyspnea, wheezing, paroxysmal cough, and chest tightness, as well as the functional demonstration of reversible airway obstruction, either spontaneously or following medical treatment [19]. 

**Figure 1:**
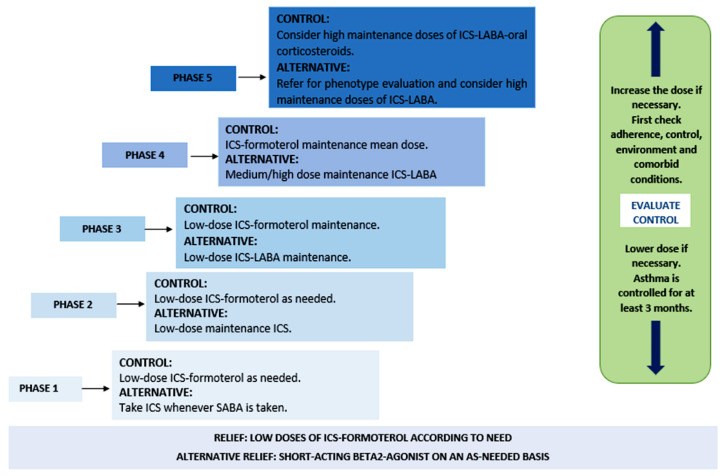
Phases of asthma treatment. Abbreviations: ICS (inhaled corticosteroids), SABA (short-acting β2-adrenergic agonists), and LABA (long-acting β2-adrenergic agonists).

Considering the manifestations of this disease, patients with asthma can receive short-term treatment for constant exacerbations of the pathology, as well as long-term treatment for its maintenance and control, as described in Figure 1 [20,21].

The prescribed anti-asthmatic medication is selected in relation to the severity of the disease and the type of asthma present [22]. There are national and international guidelines, such as those of the Global Initiative for Asthma (GINA), the National Asthma Education and Prevention Program (NAEPP), and the British Thoracic Society (BTS) [23,24], which provide recommendations on the diagnosis and treatment of asthmatic patients on an individualized basis by considering not only the severity of the disease, but also the phenotypic characteristics of the patient [2]. 

Drug therapy is mainly focused on two groups: bronchodilators and anti-inflammatory drugs. These are used to improve the patient’s symptoms by relaxing the muscles that surround the airways making it easier to breathe. The administration method used for these drugs is dependent on the state of severity present in each patient [25,26]. 

However, most of the drugs are administered through inhalers or nebulizers [27]; therefore, it is impossible to avoid contact of the drug with hard dental tissues and the oral mucosa, inducing a higher risk of adverse effects, such as dental caries, dental erosion, periodontal disease, tooth loss, bone resorption, or oral candidiasis, following prolonged use [28,29]. These effects lead to decreases in the salivary pH and flow, thus reducing the protective effects of saliva, and increases the presence of *Streptococcus mutans* and *Lactobacillus acidophilus,* which increases an individual’s susceptibility to the development of oral pathologies [30,31,32].

The adverse effects generated by medications prescribed for asthma have been the subject of research for a long time, due to the appearance of oral and dental alterations [33]. Therefore, the objective of this review is to describe the adverse effects produced by anti-asthmatic medications on dental health, based on the reported scientific evidence.

## 2. Materials and Methods

This study was developed in response to the need to obtain information in the dental field, so a bibliographic search of scientific articles published between 2002 and 2022 was carried out in the Web of Science, Scopus, and ScienceDirect databases. 

### 2.1. Bibliographic Search

We used search strategy terms indexed in the MeSH keyword health descriptor, such as “asthma”, “asthma; dentistry”, “asthma medication; dentistry”, and “asthma; oral health” to identify relevant studies in the databases indicated.

### 2.2. Inclusion Criteria

Studies reporting on the relationship between asthma and oral disorders and peer-reviewed articles written in English were included. No studies were excluded because of their design.

### 2.3. Exclusion Criteria

Studies that were not relevant to the review include articles written in Spanish, Chinese, or Russian, or gray literature. In addition, duplicate studies and studies with incomplete information, such as a lack of information on the drug used, were excluded. Likewise, information from letters to the editor, congress abstracts, and non-peer-reviewed trial registries were not included.

### 2.4. Data Analysis

In the initial search, 245 articles related to this work were identified, and after a rigorous analysis and according to the inclusion criteria, only 29 were accepted to be part of the present review.

## 3. Results

**Figure 2:**
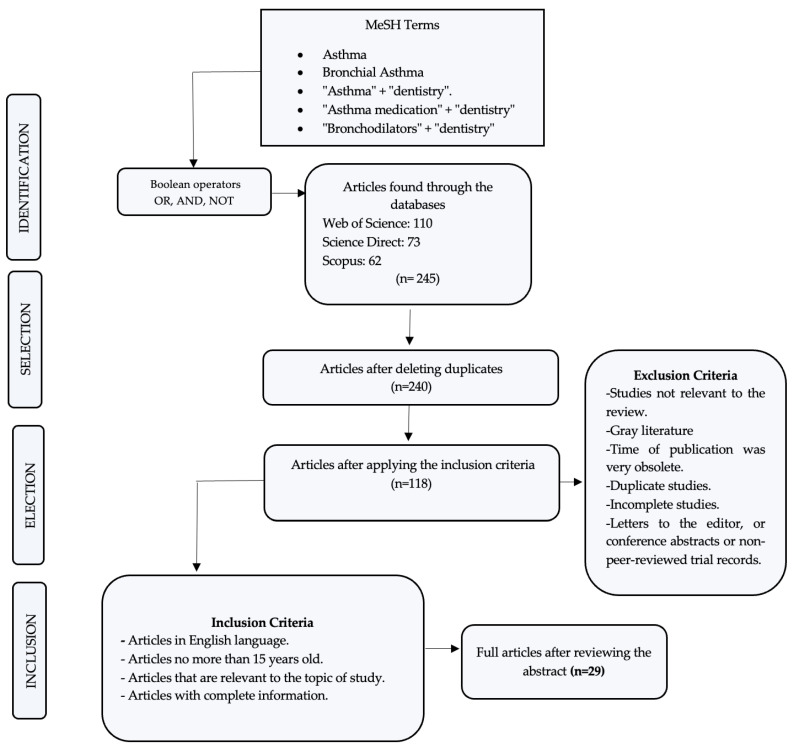
Search flow diagram.

Based on the described methodology and the scientific evidence, Figure 2 represents the PRISMA flow diagram, with the results obtained in this review.

### 3.1. Effects on Dental Health

The association of asthma with adverse effects on the dental health, such as dental caries, periodontal disease, dental erosion, and alterations at the level of the oral mucosa, has been a subject of debate among dentists [34]. Anti-asthmatic medications, especially inhalers that maintain intimate contact with oral tissues, contain fermentable carbohydrates, such as lactose monohydrate, used with the purpose of improving their taste and thus increasing the patient’s tolerance to them. Their frequent use can cause, in most cases, a decrease in salivary flow. Studies indicate that prolonged use of β2-adrenergic agonists can reduce the salivary secretion rate of the parotid gland by 36%. Therefore, as the salivary flow decreases, the levels of salivary amylase, salivary peroxidase, lysozyme, and immunoglobulin A (IgA-S) decrease [2,30,35,36].

Saliva is composed of water and a variety of inorganic and organic components, which aid in the masticatory process and protect dental structures from existing pathogenic microorganisms. Therefore, the salivary composition plays an important role in oral health, as it acts as a defensive mechanism. In addition, anti-asthmatic medications are frequently administered at night before going to sleep without performing any routine method of oral hygiene, which could increase the risk of alterations at the level of the oral cavity [2,37,38,39].

Table 1 summarizes the studies found on the adverse effects of anti-asthmatic drugs in the oral cavity. The studies are ordered by year of publication, type of study, parameters tested, drugs analyzed, study design, and most relevant results.

There are several international medical guidelines that establish the objectives for optimizing therapy in asthmatic patients, such as preventing and minimizing exacerbations of chronic and acute symptoms, and the requirement for emergency care. Several investigations have shown that many patients can achieve good asthma management through long-term control treatment, mainly with inhaled corticosteroids (ICS), long-acting β2-adrenergic agonists (LABA), and short-acting β2-adrenergic agonists (SABA) [4,42,60,61,62,63]. However, the increased presence of side effects that accompany the prolonged use of these medications should be considered [26,48,64]. Inhaled or nebulized SABAs resolve acute asthma symptoms, and their therapeutic effect occurs during the first 15 to 20 min of the first hour of acute asthma, inducing symptomatic relief, but they have no effect on airway inflammation and do not provide a sustained benefit [26,51,57,65]. The inhalation route is considered the most effective method of drug administration for asthmatic patients, because it avoids the effect of the first hepatic step with a minimal reduction in bioavailability and rapid absorption of the drug from the alveolar region to the bloodstream, thus improving the therapeutic efficacy. There are three pharmaceutical presentations that allow for the drug to be administered: through an inhalation aerosol, pressurized metered-dose inhaler (MDI), or a dry powder inhaler (DPI) or nebulizer [65,66,67,68]. The management of these formulations is complex at the pediatric level, and almost 30% of asthmatic adults have an inadequate inhalation technique, triggering 80% of the drug to be deposited in the oropharynx, which considerably changes the effectiveness of the treatment and can cause local or systemic side effects [55,69]. In addition, drugs have considerable effects on salivary components and properties, especially in terms of the buffering capacity, quantity, and viscosity. It is considered that any factor that reduces the quantity and quality of saliva can negatively affect oral health, because saliva plays a very important role in its preservation [37,43].

Based on the literature found, Table 2 of the drugs administered in asthma and the adverse effects that they produce was developed. Among the main adverse effects associated with anti-asthmatic medications are oropharyngeal candidiasis, laryngeal weakness, dental caries, decreased taste, burning tongue, tongue abrasion, and a decreased bone marrow density level, as well as the other adverse effects summarized in Table 2 [32,70]. 

The main adverse effects that affect dental health produced by anti-asthmatic medications are described below.

#### 3.1.1. Asthma and Dental Caries

Dental caries is one of the oral pathologies with the highest prevalence worldwide. Thus, it is considered a public health problem. It has a prevalence of 80% in children and 100% in adults [54,74,75,76]. The appearance of carious lesions results from an alteration in the balance between dietary-bacterial factors, the components of the host, and the use of various medications [77,78]. The main treatment for asthma is the inhalation of steroids, and this is an important causal factor in the development of dental caries, because of the direct changes in the salivary composition that occur [79,80,81]. Several factors contribute to the increased prevalence of dental caries in asthmatic patients, such as a reduced saliva flow caused especially by beta-2 adrenergic agonists, increased counts of *Streptococcus mutans* and *Lactobacillus* spp., and a decreased salivary pH below the critical value 30 min after treatment with beta-2 agonist inhalers [22,36,73,82].

In addition, most anti-asthmatic medications contain fermentable carbohydrates [83]. Therefore, frequent oral inhalation of these sugar-containing drugs, coupled with decreased salivary production, may contribute to an increased risk of dental caries. Previous research has shown that the duration of medication has a significant influence on the risk of developing dental caries in asthmatic patients, indicating that patients using salbutamol inhalers are at higher risk of developing carious lesions compared with non-asthmatic patients [84,85]. Similarly, the frequency of use and dosing times influence the prevalence of carious lesions. Studies have shown that children who use this type of medication more than twice a day experience more dental caries and that this risk increases in children whose dose is administered before bedtime [2].

Therefore, it is essential to prescribe fluoride supplements to asthmatic patients, especially those taking beta-2 adrenergic agonists. Patients should be educated to perform mouth rinses after using inhalers. Chewing sugarless gum for at least one minute after drug administration will help to neutralize salivary pH, stimulate salivary flow, and buffering oral acids [2,36,52,85,86].

#### 3.1.2. Asthma and Oral Candidiasis

Oral candidiasis is the most prevalent opportunistic fungal infection at the level of the oral cavity. This fungal infection is caused by species of the genus Candida, with Candida albicans being the most common species, representing more than 80% of clinical isolates; however, there are other responsible species, such as *C. tropicalis*, *C. glabrata*, *C. krusei*, and *C. parapsilosis*, that enhance the resistance to antifungal therapy [52,87,88]. Pathological changes in the oral mucosal surface, immunological changes, and microenvironmental variations contribute to the development of this opportunistic infection [52,87]. Thus, local and systemic factors, such as decreased salivary flow, erosion of the oral mucosa, vitamin deficiency, and generalized immunosuppression play important roles in the etiology of this lesion [52].

There are certain population groups that are prone to the development of oral candidiasis, such as patients being treated with systemic immunosuppressants or topical steroids, those with a dry oral mucosa, and those with anemia, among other systemic conditions [52]. It has been highlighted that the regular use of inhaled corticosteroids in asthmatic patients is associated with the development of oral candidiasis, the prevalence of this infection as a possible adverse effect varies from 0 to 77% [89]. The relationship between the administration of inhaled corticosteroids and candidiasis is mainly due to the decreases in salivary IgA and histamine due to the actions of immunosuppressants, considering that only 20% of the inhaled dose actually reaches the lungs, so most of the inhaled drug remains in the oral cavity and oropharynx, affecting the physiology of the oral tissues, in addition to suppressing cellular immunity and phagocytosis. However, it has been reported that oral mucosal immunity normalizes upon discontinuation of corticosteroids [50,59,89,90,91,92].

To reduce the incidence of oral candidiasis in patients undergoing asthma treatment, it is recommended that mouth rinses are performed immediately after the administration of the drug and before going to sleep. For this purpose, rinses based on 0.05% neutral sodium fluoride or with an antimicrobial base can be used. It has even been shown that gargling with a diluted amphotericin solution considerably reduces the amount of Candida albicans in relation to gargling with water. In addition, regular dental check-ups are advised [89]. The administration of topical antifungals, such as nystatin, and medications that promote salivary flow, such as sugar-free chewing gum, are also recommended [93]. 

#### 3.1.3. Asthma and Periodontal Disease

Periodontal diseases represent complex interactions between host defenses and bacterial pathogen aggression that result in inflammatory processes in the dental supporting tissues. Signs and symptoms may vary: from gingivitis, which refers to inflammation of the gingival tissues, to periodontitis, in which an inflammatory process of the supporting tissues occurs, involving clinical attachment loss and bone loss, possibly resulting in tooth loss [94,95]. Periodontal health appears to be strongly related to an adequate salivary flow. One of the most effective elements in the defense of the oral cavity is saliva, which has antibacterial, antiviral, and antifungal activity, as well as containing immunoglobulins and enzymes that play protective roles for the mucosa. Therefore, a decrease in the salivary flow directly affects periodontal tissues, and it has been reported that anti-asthmatic medications cause alterations to salivary secretion, negatively influencing periodontal health [96,97,98,99].

Therefore, the main cause of periodontal tissue deterioration in asthmatic patients is the reduction of the protective action of saliva. Another factor that contributes to the presence of these alterations is the tendency of these patients to breathe through the mouth, thus causing continuous dehydration of the alveolar mucosa, especially during an acute asthmatic attack [100,101,102]. They also have a higher prevalence of calculus due to elevated levels of calcium and phosphorus [85], and these patients may have high IgE levels in the gingival tissues, a factor that is also directly related to poor periodontal health. Previous research indicated that diseases other than asthma have direct relationships with periodontal disease [2,36], such as allergic rhinitis, obstructive sleep apnea, and adenoid and tonsil hypertrophy, among other respiratory diseases that favor mouth breathing [2].

#### 3.1.4. Asthma and Dental Erosion

Dental erosion is the progressive and irreversible erosion of mineralized dental tissues due to chemical processes without bacterial involvement [78,103], an example being anti-asthmatic medications that can contribute to the development of this non carious lesion by reducing salivary protection against extrinsic and intrinsic acids [104,105,106]. In addition, the main drugs used to treat asthma, especially those in powder form, can lead to a pH below 5.5 [40,41]. Similarly, beta-2 adrenergic agonists can cause the relaxation of other smooth muscles, such as the lower esophageal sphincter, and increase the amplitude of esophageal contraction, leading to symptoms, such as gastric reflux. Therefore, asthmatic patients tend to present with dental erosion due to constant acid attacks.

It is essential to instruct asthmatic patients to rinse their mouths with sodium bicarbonate or neutral sodium fluoride after using inhalers [104]. Similarly, these patients should be instructed that it is not advisable to brush their teeth immediately after exposure to acids, as this may further damage the already weakened enamel. Another measure that helps to reduce erosion rates in asthmatic subjects is the use of a spacer device during the administration of dry powder inhalers, these spacers consist of plastic devices that minimize the contact between the drug and the oropharynx, meaning that most of the drug is directed towards the lungs [2,17,36].

## 4. Discussion

Thomas et al. [36] pointed out that most asthma medications are significantly associated with alterations in oral health, and patients undergoing anti-asthma treatment should be instructed on the use of their prescribed inhalers, since constant and direct contact between the formulations and the oral cavity increases the risk of oral tissue affection and, therefore, the development of pathologies. Some dry powder inhalers are known to contain sugar to increase the tolerability of the taste of the drug when administered. Frequent oral inhalation of sugar combined with a reduced salivary flow and decreased saliva pH may contribute to an increase in dental caries [80]. Stensson et al. [77] explained that this association may be due to the presence of fermentable carbohydrates, such as lactose monohydrate, in anti-asthmatic medications. These excipients may increase the risk of oral conditions, such as dental caries, by acidifying the pH of the oral cavity due to acid formation from the fermentation of ingested carbohydrates. Shashikiran et al. [85] demonstrated that patients who use bronchodilators, such as salbutamol, are more prone to the development of dental caries in comparison with those taking other anti-asthmatic medications. Likewise, the authors stated that patients who consume beta-2 adrenergic agonists also present with a high incidence of dental caries due to a reduction of salivary flow and an increase in the proliferation of cariogenic microorganisms. Moreover, after investigating oral health indices in healthy children and children with mild to moderate asthma, Ehsani et al. [44], found that there were no significant differences between children with asthma and those without asthma with respect to the DMTF index (decayed, missing, filled teeth) with means of 3.34 in the asthmatic children and 3.0 in the control group. In the study, Brigic et al. [31] reported the cariogenic potential of inhaled anti-asthmatic medications, observing that non-asthmatic subjects presented with a significantly higher DMTF index than asthmatic subjects (*p* = 0.004). We also determined that there were no significant differences in the values of the plaque index between groups (*p* > 0.05).

Among the anti-asthmatic medications, it has been shown that high doses and prolonged duration of inhaled medications are closely related to adverse effects on oral tissues. Manuel et al. [104], reported that inhalers containing salbutamol and fluticasone propionate decrease salivary flow 30 min after inhalation of the drug, indicating that higher bronchodilator doses are associated with greater reductions in the levels of immunoglobulin A (IgA), calcium, lactoferrin, and total protein in the saliva, so that patients who do not benefit from the protective role of saliva are more vulnerable to the development of oral pathologies.

Another adverse effect caused by the consumption of anti-asthmatic medications is oral candidiasis. The growth of *Candida* in asthmatic patients is very high compared to healthy people. Anti-asthmatic medication makes the oral habitat prone to attack from opportunistic infections, such as oral candidiasis. [59]. Moreover, Lemmetyinen et al. [92] showed that one of the conditions related to asthma drugs is a viral infection. The authors found that the rate of herpes zoster was almost seven times higher in asthmatic subjects than in non-asthmatic subjects. The authors pointed out that this may be because asthmatic patients have an immune system deficiency, which may cause reactivation of the varicella zoster virus.

Gani et al. [2] pointed out that dental erosion is associated with the use of β2-adrenergic agonists, regardless of the type of inhaler used, but cofactors, such as lifestyle, a diet rich in acids, or diseases that cause abnormal relaxation of the lower esophageal sphincter muscle, can increase the risk of dental erosion. It has even been reported that this abnormal relaxation of the muscle can be associated with the use of theophylline. Shashikiran et al. [85] conducted a case-control study on patients from Southeast Queensland and found higher incidences of dental hypersensitivity, xerostomia, salivary gland abnormalities, gastric discomfort, and self-induced vomiting in asthmatic patients compared to non-asthmatic patients. They explained that asthmatics are at risk of dental erosion due to the extrinsic acid to which they are constantly exposed.

Mappangara et al. [102] stated that the use of corticosteroid anti-asthmatic medications, especially via inhaled methods, increases the risk of periodontal diseases, such as gingivitis and severe periodontitis. The immunosuppressive effect of corticosteroids may influence the periodontal tissue response. These agents inhibit the host response, resulting in the clinical expression of gingivitis, [2] thus demonstrating that bronchodilators administered by the inhalation route are the anti-asthmatic medications that cause the most adverse effects in the oral cavity.

### 4.1. Perspectives

Respiratory problems nowadays have reached a high percentage, especially in the pediatric population, hence the imperative need to study the anti-asthmatic medications used for their treatment; there are reports that the continuous use of these drugs is related to the generation of other pathologies. In the dental area, they are directly related to an increase in the percentage of dental caries, the presence of periodontal and gingival disease, tooth loss due to changes in the salivary composition, and pH. Therefore, this review seeks to highlight the importance of adverse effects on dental health to provide effective pharmacotherapeutic follow-up that largely prevents dental damage. It is necessary to mention that there are preventive measures to avoid adverse effects on dental health, such as adequate hygiene after the application of medications, and this is where the contribution of the patient is very significant, because it will allow to control and reduce the incidence of adverse effects in the oral cavity.

### 4.2. Strengths

Among the strengths found in this study is the mention of the importance of knowing the different adverse effects that occur after administering anti-asthmatics at the oral level, and we hope that through this collection of information, we can begin to investigate these consequences in depth, so that the health professional can see the issues and can solve them.

### 4.3. Limitations of the Study

Among the limitations, the lack of specific information on the relationship between the prescription of anti-asthmatics and the dental area is indicated, which promotes further research in this area. Added to this is the scarce information on the indication, dosage, and presentation of the drug in the different publications revised in this article.

## 5. Conclusions

The use of anti-asthmatic medications can lead to a series of oral alterations due to an imbalance in the protective factors associated with the oral cavity, which can cause diseases, such as dental caries, dental erosion, tooth loss, periodontal disease, bone resorption, as well as fungal infections, such as oral candidiasis. These alterations are mainly due to the decrease in salivary flow, which leads to a reduction in the protection of the oral cavity by saliva due to reduced levels of defense components, such as IgA, calcium, and lactoferrin, among others. Inhalation therapy is closely related to the production of these adverse effects due to the direct contact of the drug with the oral cavity and oropharynx, so it is essential to educate asthmatic patients about preventive measures, such as mouth rinses with sodium bicarbonate or neutral sodium fluoride after using inhalers; chewing sugarless gum for at least one minute after drug administration to help neutralize the salivary pH, stimulate salivary flow, and buffer oral acids; or even the use of a spacer device during the administration of dry powder inhalers to minimize contact between the drug and the oropharynx.

## Figures and Tables

**Table 1:** Characteristics of the included studies.

Type of Study	Author/Year	Objetives	Drugs	Study Design	Main Findings	References
Case—control	Sivasithamparam et al./2002	To determine the incidence of dental erosion in asthmatic and non-asthmatic patients.	Salbutamol	Subjects were examined by recording tooth wear patterns.	There was a higher incidence of dental hypersensitivity, xerostomia, salivary gland abnormalities, gastric discomfort, and self-induced vomiting in asthmatic patients compared to the control group.	[40]
Retrospective study	Dugmore et al./2004	To analyze the relationship of possible etiological factors for the presence of dental erosion.	Terbuline Salbutamol	The erosion index used was based on the 1993 Children’s Dental Health Survey.	At 12 years, significant positive associations were found between the experience of erosion and decay (odds ratio [OR] = 1.48), drinking fruit juice (OR = 1.42) or soft drinks (OR = 1.59–2.52, depending on quantity and frequency).	[41]
Case—control	Ersin et al./2006	To investigate the dental caries risk of asthmatics in relation to dental plaque indices, salivary flow rate, pH and buffering capacity, saliva composition, and salivary levels of Streptococcus mutans compared to healthy subjects.	Inhaled corticosteroids B2 agonists Leukotrienes	The World Health Organization criteria were used for dental examinations and the Silness and Löe plaque index was used for plaque indices.	Children in the asthmatic group aged 6 to 10 years had a significantly higher prevalence of dental caries compared to the control group of the same age.	[33]
Randomized clinical trial	Huchon et al./2009	To evaluate lung function and asthma control with beclomethasone and formoterol in a single inhaler.	Beclamoentasone/formoterol inhaled	Patients received extrafine fixed combination treatment of beclomethasone dipropionate 200 mg/formoterol 12 mg twice daily, or beclomethasone dipropionate and formoterol.	The combination of beclomethasone dipropionate/formoterol through a single inhaler or through separate inhalers improved morning lung function.	[42]
Case—control	Botello et al./2011	To evaluate the dental caries risk of asthmatic patients based on the levels of *Streptococcus mutans* and *lactobacillus* in saliva samples, as well as the index of oral hygiene and dental caries.	Beclomethasone Budesonide Ciclesonide Fluticasone propionate Mometasone furoate	Parents were interviewed about factors related to oral health. WHO criteria for dental examinations were used.	No differences were observed between the asthma and control groups for the prevalence of dental caries in children aged 3 to 6 years and 7 to 10 years, except for severe cases in the younger group.	[32]
Cross-sectional study	Stensson et al./2011	To study oral health in young adults with long-term controlled asthma.	Salmeterol/Fluctisona Formoterol/budenoside	A clinical examination was performed to determine the prevalences of dental caries, erosions, gingival inflammation, cervicular fluid, and presence of periodontal pockets and the rate of plaque formation.	The asthmatic participant group had more gingivitis (*p* = 0.01) and a lower stimulated salivary secretion rate than the controls (*p* = 0.01). Asthmatics also had a somewhat lower initial pH value, although not statistically significant.	[34]
Case—control	Boskabady et al./2012	To examine the relationship between type of inhaled medication, duration of use, dosage, technique of inhaler use, and severity and duration of disease on dental caries in asthmatic patients.	Corticosteroids β2-adrenergic receptor agonists	In asthmatic patients, the type, dose, duration of medication, technique of inhaler use, and severity and duration of the disease were recorded. Dental health status, including DMFT index, was examined. In addition, pulmonary function tests (PFTs) were performed for both groups.	All dental cavity rates in the asthmatic group were higher than those of the controls. There was no significant correlation between dental cavity rates and disease duration, PFT values; medication dose or inhaler use technique.	[43]
Case—control	Ehsani et al./2013	To investigate oral health indices in healthy children and children with mild to moderate asthma.	Salbutamol Inhaled corticosteroids Fluctisone Beclomentasone Systemic corticosteroids Antihistamines Theophylline	Dental plaque, gingival inflammation, mouth breathing and dental caries were assessed by a trained examiner according to the World Health Organization criteria.	The results indicated no significant differences between the children with asthma and those without asthma regarding (decayed, missing, filled, teeth) dmft index (mean of 3.34 in asthmatic children and 3.0 in the control group).	[44]
Case—control	Godara et al./2013	To examine the potential link between dental caries and the specific use of dust inhalers in patients with bronchial asthma.	B2 agonists Inhaled corticosteroids	A clinical examination performed, which included the DMFT index to assess the presence of dental caries.	Asthmatic subjects exhibited a higher incidence of dental caries compared to the control group, but the difference was not statistically significant.	[45]
Case—control	Alaki et al./2013	To investigate the prevalence and severity of dental caries in children with a history of asthma.	Inhaled corticosteroids β2-agonist inhalers Anti-asthma 2 agonist inhalers and antihistamines combined with corticosteroids	Interviews and questionnaires were completed by the parents of the children involved and dental examinations were performed. Stimulated salivary samples were collected to determine salivary flow rate, buffering capacity, and salivary levels of *Streptoccocus mutans* and *Lactobacillus*.	No significant differences were found in the DMFT index or community periodontal index scores between cases and controls.	[19]
Case—control	Prasanthi et al./2014	Evaluation of the effect of diuretics on oral health status with respect to pH, buffering, total protein content, various ion concentrations, and oral mucosal lesions.	Agonists of β2-adrenergic receptors.	Unstimulated and stimulated saliva was collected for further evaluation. Dental caries and periodontal status were measured using the DMFT index and Russell’s periodontal index, respectively.	Prevalence was found for dental caries (*p* < 0.01), periodontal status (*p* < 0.001), and mucosal lesions (*p* < 0.01).	[46]
Case—control	Monadi et al./2015	To investigate the effect of inhaled corticosteroids on bone mineral density.	Inhaled corticosteroids	Bone mineral density in the lumbar spine (LS) and femoral neck (FN) was measured by dual energy X-ray absorptiometry (DEXA).	Compared to controls, bone mineral density decreased only in patients < 50 years in both spine (11.3%, *p* = 0.013) and hip (8.8%, *p* = 0.044), while in patients ≥ 50 years, BMD did not decrease significantly.	[47]
Retrospective-prospective study	Brigic et al./2015	Cariogenic potential of inhaled anti-asthmatics	Inhaled anti-asthmatics	200 patients, aged 7 to 14 years, divided into two groups: the control group (n1 = 100) was made up of healthy children and the experimental group was made up of children suffering from asthma (n2 = 100). In both groups of respondents, the following were found inthe DMFT index: the plaque index value and the hygienic-dietary habits by means of the questionnaire.	The subjects in the control group had a significantly higher DMFT index than the subjects in the experimental group (*p* = 0.004). It is determined that there are no significant differences in plaque index values (*p* > 0.05).	[31]
Retrospective-prospective study	Brigic et al./2015	Asthma/dental caries	Inhaled anti-asthmatics	The study sample consisted of 200 patients, aged from 7–14 years, divided into two groups: the control group (n1 = 100) consisted of healthy children and the experimental group consisted of children suffering from asthma (n2 = 100). In both groups of respondents, the following were determined in the DMFT index: plaque index value and hygienic-dietary habits using the questionnaire.	The results of this research have once again confirmed that the dental caries is a disease with multifactorial etiology in which mutual relationships and interactions between numerous etiological factors contribute even more to the complexity to estimate the of cavity risk problem.	[30]
Randomized clinical trial	Reddel et al./2017	To analyze the benefits of initiation of inhaled corticosteroid therapy for mild asthma on symptom frequency.	Inhaled corticosteroids	Study of treatment with inhaled steroids as regular therapy (START) of 3 years, conducted in 32 countries, with clinical visits every 3 months.	Of 7138 patients (*n* = 3577 budesonide; *n* = 3561 placebo), the initial frequency of symptoms was 0 to 1 days per week for 2184 (31%) participants, more than 1 and less than or equal to 2 days of symptoms per week for 1914 (27%) participants, and more than 2 days with symptoms per week for 3040 (43%) participants.	[48]
Case—control	Arafa et al./2017	To evaluate oral health status and salivary composition in a group of children with bronchial asthma.	Inhaled corticosteroids β2-agonist inhalers	The children were clinically examined to assess their dental cavity experience, dental erosion status, and gingival status.	The results of this study revealed that asthmatic children presented a significantly higher mean DMFT index, dental erosion status, and gingival status compared to the control groups.	[27]
Case—control	Rodríguez et al./2018	To determine whether oral health is a risk factor for community-acquired pneumonia in asthmatic patients undergoing inhaled therapy.	Inhaled anti-asthmatics	The main study factor was the general oral health assessment index (GOHAI) score.	Bivariate analysis shows a statistically significant association of community-acquired pneumonia with a GOHAI ≤ 57 points (poor oral health) (OR 1.69).	[49]
Case—control	Ashuja et al./2018	To evaluate and compare *Streptococcus mutans* (SM) and plaque lactobacilli and *C. albicans* counts in saliva samples from asthmatic adults with controls during the course of medication longitudinally.	Budesonide Methylprednisolone Formoterol	Samples were collected from twenty newly diagnosed asthmatic adults and twenty controls for estimation of microbial counts at baseline and at three and six months after initiation of medication.	Asthmatics at baseline had higher microbial counts than controls, but the difference was not statistically significant.	[50]
Cohort	Wu et al./2019	Asthma/caries	Inhaled corticosteroids Bronchodilator agents: short or long-acting b2 agonists	Investigating the correlation between asthma medications and dental caries among children in Taiwan.	The prevalence of caries in children without asthma was 85.2% and that of children with asthma was 90.0%, children who received asthma medication have a higher prevalence of dental caries and a higher rate of severe caries than children without asthma.	[22]
Cross-sectional study	Rezende et al./2019	To evaluate caries, erosion, and enamel defects in children with and without asthma.	Salbutamol	The assessment consisted of an oral examination and a structured interview with the children’s parents/guardians.	Of 112 asthmatic children, 63 (51.2%) had dental caries and 25 (53.2%) had enamel defects. In the adjusted analysis, dental caries and salbutamol use were associated (PR = 1.32, 95% CI = 1.01–1.72).	[39]
Retrospective study	Hu et al./2019	To assess the characterization of oral candidiasis and the species profiles in such patients.	Corticosteroids, vasoconstrictors, and antihistamines	Over a period of four consecutive years, patients with oral mucosal diseases were screened for oral candidiasis through a combination of clinical presentation and laboratory findings.	In total, 9769 (6.09%) of the 160,357 patients examined were diagnosed as having oral candidiasis on the basis of both clinical manifestations and laboratory tests.	[51]
Case—control	Hassanpour et al./2019	To determine and compare the frequency of dental caries among asthmatic children, with asthmatic children who inhaled corticosteroid treatment and healthy children.	Inhaled corticosteroids	An examination for the diagnosis of dental caries was performed using the DMF index.	The mean DMF index score in each of the subscales of decayed teeth (D/d), missing teeth (P/p), and filled teeth (O/o), was higher in asthmatic children compared to healthy children (*p* < 0.05).	[52]
Case—control	Khassawneh et al./2019	To study the association between bronchial asthma (BA) and periodontitis.	Oral corticosteroids and inhaled corticosteroids	AB cases were diagnosed by a physician and subjects had been prescribed anti-asthma medications for ≥12 months. Periodontitis was defined as the presence of ≥4 teeth with ≥1 site with probing depth (PPD) ≥ 4 mm and clinical attachment level (CAL) ≥ 3 mm.	Periodontitis was present in 52 (40.0%) patients with AB and 26 (20.0%) in the control group, *p* < 0.005.	[53]
Case—control	Chumpitaz-Cerrate, et al./2020	To determine the prevalence of dental caries in pediatric asthmatic patients using inhaled drugs.	Budesonide/salbutamol or fluticasone/salmeterol	A medical examination was performed to determine the type, timing, and frequency of treatment and an oral examination to establish the prevalence of dental caries and the decayed, missing, and filled tooth DMFT index.	The prevalence of dental caries was 34.2% in the control group and 28.3% in the case group (*p* = 0.094). In relation to the rate of dental caries, the DMFT index in the control group was 4.73 ± 0.32 and in the case group 3.98 ± 0.31 (*p* = 0.08).	[54]
Cross-sectional comparative study	Bairappan et al./2020	To assess and compare the salivary characteristics and oral health, and to evaluate the impact of asthma and its medication on dental caries among adolescents with and without asthma.	Short-acting beta-agonists, systemic corticosteroids, and anticholinergic drugs	Study was conducted among 50 asthmatic and 50 nonasthmatic adolescents aged 12–15 years. Salivary samples were collected to determine the flow rate, pH, buffering capacity, and Streptococcus mutans and Lactobacilli counts. Oral health assessment was performed using WHO 2013 proforma.	Asthmatic participants had a significantly higher mean number of teeth with dental caries, gingival bleeding, and dental erosion than nonasthmatics (*p* < 0.05). The prevalence of fluorosis, traumatic dental injuries, and oral mucosal lesions in asthmatics were 34.0%, 38.0%, and 28.0%, respectively. Statistically significant difference was found in the flow rate, pH, buffering capacity, S. mutans and Lactobacilli counts, and decayed, missing, filled teeth (DMFT) index between asthmatic and nonasthmatic participants.	[55]
Cross-sectional study	Brasil-Oliveira et al./2021	To assess oral health-related quality of life (OHRQoL) among individuals with severe asthma, comparing it with that observed among persons with mild to moderate asthma and persons without asthma.	Inhaled corticosteroids	The index of decayed, missing, and filled teeth (DMFT index) was calculated, as well as the periodontal screening and recording index, and salivary flow was determined.	Periodontitis and reduced salivary flow were more common in patients with severe asthma compared to the group of participants with mild to moderate asthma and no asthma.	[56]
Cross-sectional study	Akiki et al./2021	To assess the prevalence of physician-diagnosed asthma and current asthma, and its determinants.	Inhaled corticosteroids, and antihistamines	The questionnaire used collected information on asthma, respiratory symptoms, and risk factors.	The prevalence of physician-diagnosed asthma was 6.7% (95% CI 5–8.7%) and that of current asthma was 5% (95% CI 3.6–6.9%).	[57]
Meta analysis	Slob, et al./2021	To evaluate whether genetic variations were associated with exacerbations in children treated with beta2-agonists from a global consortium.	Long-acting beta2-agonist	To analyze the genome-wide association performed in 1425 children and young adults with asthma (age 6–21 years) withregular use of beta2-agonists.	Genome-wide association results were analyzed for a total of 82,996 common single nucleotide polymorphisms (SNPs, MAF ≥ 1%) with high imputation quality. Eight independent variants were suggestively (*p*-value threshold ≤ 5 × 10^–6^) associated with exacerbations despite beta2-agonist use.	[58]
Prospective study	Abidullah et al./2022	This study aimed to estimate salivary Candida Albicans in asthmatic patients taking anti-asthmatics medication.	Anti-asthmatic medicine in doses of 100, 250, or 500 mg	The research comprised a total of 100 individuals, 50 of whom were asthmatics, and 50 healthy controls who were age and sex-matched to the asthmatics.	32 people had candida growth and 18 individuals did not have any candidal development at all. Eighteen people were in the 400 CFU/mL group, and 32 individuals were in the 401 CFU/mL group, respectively. It was 0.000 in the 400 colony forming unit/milliliter group, and 27,200 in the 401 CFU/mL group, with 0.00 being the median. There was a notable difference between study and control groups in terms of colony forming unit per milliliter (*p* = 0.000).	[59]

**Table 2:** Medications for asthma and adverse effects.

Group	Type of Medication	Drugs	Mechanisms of Action	Indications	Adverse Effects	Reference
β2 Beta-adress beta agonists inhalation bronchodilators	Short-acting β2-adrenergic agonists (SABA)	Terbutaline Fenoterol Salbutamol Albuterol	Relaxes smooth muscles, activates b2 adrenergic receptors in the lungs, bronchodilation. Duration of 2 to 3 min after administration.	Indicated for the rapid relief and prevention of bronchospasms caused by physical exercise.	Oropharyngeal deposition by pharmaceutical inhalers: 60–70%. Decreased buffering capacity of saliva. Increased cariogenic bacterial load in children. Dental erosion, candidiasis, gingivitis. Acid oral pH favoring the production of cariogenic bacteria. Alteration of salivary composition. Susceptibility to dental caries.	[43,45,67,68,71,72,73]
	Long-acting β2-adrenergic agonists (LABAs)	Indacaterol, Salmeterol, Formoterol Olodaterol Vilanterol Arfomoterol	Duration about 12 h.			
Anticholinergics	Bronchodilators	Ipratropium Bromide	Antimuscarinic agent, decrease in smooth muscle contractility resulting in bronchodilatation.	Moderate to severe exacerbations (emergencies). Transient pupil dilation and blurred vision.	Dry mouth, intestinal constipation, blurred vision, aggravation of glaucoma.	
Corticosteroids	Intermediate and long duration	Prednisone Dexamentasone Blecomethasone Methylprednisolone	Anti-inflammatory; reverses B2 receptor down-regulation.	Reduces inflammatory symptoms indicated for the treatment of moderate to severe exacerbations.	Oropharyngeal deposition by pharmaceutical inhalers: 60–70%. Decreased buffering capacity of saliva. Increased cariogenic bacterial load in children. Dental erosion, candidiasis, gingivitis. Acid oral pH favoring the production of cariogenic bacteria. Alteration of salivary composition. Susceptibility to dental caries.	
	Inhaled	Ciclesonide Fluticasone Fluorosone propionate Fluticasone propionate Mumetasone fluorosideBudesonide	They inhibit migration, activation of inflammatory cells and secretion of proinflammatory substances.			
Antihistamines, Anti H1 Antihistamines, H1 Receptor Antagonists		Ketotifen Diphenhydramine	They stabilize membranes and block the release of mediators, reducing epithelial damage.	Restricted corticosteroid use, moderate exacerbations in children.	Central nervous system depression or paradoxical CNS stimulation, dry mouth, dizziness and convulsions.	
Chromoglycate		Sodium chromoglycate	Reduction of bronchial hyperresponsiveness, anti-inflammation.	Preventive treatment, exercise-induced bronchospasm.	Mild pharyngeal irritation, transient cough and bronchospasm may occur.	
Teofilina		Theophylline	Bronchodilator for systemic use, relaxing the smooth muscles of the bronchial tree and pulmonary vessels.	Preventing and treating wheezing, shortness of breath, and chest tightness.	Symptoms and signs of central nervous system and cardiovascular system stimulation, at high doses. Nausea, gastroesophageal reflux, vomiting, headache, insomnia, nervousness, tachycardia, tremor, in rare doses.	

## Data Availability

Not applicable.

## Abbreviations

GINA	Global Initiative for Asthma
NAEPP	National Asthma Education and Prevention Program
BTS	British Thoracic Society
WHO	World Health Organization
ICS	Inhaled corticosteroids
LABA	Long-acting β2-adrenergic agonists
SABA	Short-acting β2-adrenergic agonists
MDI	Pressurized metered-dose inhaler
DPI	Dry power inhaler
SIg-A	Secreted immunoglobulin A
IgA	Immunoglobulin A
DMTF	Decayed, Missing, and Filled Teeth index
PFT	Pulmonary function tests
OH: SALSA	Health Oral San Antonio Longitudinal Study of Aging
LS	Lumbar spine
FN	Femoral neck
DEXA	Dual energy X-ray absorptiometry
BMD	Bone mineral density
PPD	Probing depth
CAL	Clinical attachment level.
START	Steroids as regular therapy.
GOHAI	General Oral Health Assessment Index.
